# Autophagy plays a protective role against *Trypanosoma cruzi* infection in mice

**DOI:** 10.1080/21505594.2019.1584027

**Published:** 2019-03-04

**Authors:** Ana Florencia Casassa, María Cristina Vanrell, María Isabel Colombo, Roberta A. Gottlieb, Patricia Silvia Romano

**Affiliations:** a Laboratorio de Biología de Trypanosoma cruzi y la célula hospedadora- Instituto de Histología y Embriología “Dr. Mario H. Burgos”, (IHEM-CONICET- Universidad Nacional de Cuyo), Mendoza, Argentina; b Facultad de Ciencias Médicas, Universidad Nacional de Cuyo, Mendoza, Argentina; c Laboratorio: Mecanismos moleculares implicados en el tráfico vesicular y la vía autofágica Instituto de Histología y Embriología (IHEM) “Dr. Mario H. Burgos”, (IHEM-CONICET- Universidad Nacional de Cuyo), Mendoza, Argentina; d Smidt Heart Institute, Cedars-Sinai Medical Center, Los Angeles, CA, USA

**Keywords:** Autophagy, xenophagy, *T. cruzi* infection, Beclin-1, Beclin-1 heterozygous knockout mice

## Abstract

Autophagy is a catabolic pathway required for cellular and organism homeostasis. Autophagy participates in the innate and adaptive immune responses at different levels. Xenophagy is a class of selective autophagy that involves the elimination of intracellular pathogens. *Trypanosoma cruzi* is the causative agent of Chagas, a disease that affects 8 million individuals worldwide. Previously, our group has demonstrated that autophagy participates in the invasion of *T. cruzi* in non-phagocytic cells. In this work we have studied the involvement of autophagy in the development of *T. cruzi* infection in mice. Beclin-1 is a protein essential for autophagy, required for autophagosome biogenesis and maturation. We have performed an acute model of infection on the autophagic deficient *Beclin-1* heterozygous knock-out mice (Bcln^±^) and compared to control Bcln^+/+^ animals. In addition, we have analyzed the infection process in both peritoneal cells and RAW macrophages. Our results have shown that the infection was more aggressive in the autophagy-deficient mice, which displayed higher numbers of parasitemia, heart´s parasitic nests and mortality rates. We have also found that peritoneal cells derived from Bcln^±^ animals and RAW macrophages treated with autophagy inhibitors displayed higher levels of infection compared to controls. Interestingly, free cytosolic parasites recruited LC3 protein and other markers of xenophagy in control compared to autophagy-deficient cells. Taken together, these data suggest that autophagy plays a protective role against *T. cruzi* infection in mice, xenophagy being one of the processes activated as part of the repertoire of immune responses generated by the host.

## Introduction

Eukaryotic cells have three main vesicular pathways for degradation. Endocytosis and phagocytosis are involved in the lysis of extracellular proteins and microorganisms, respectively, whereas autophagy is a self-degradation pathway wich works to remove intracellular components such as long-lived proteins and old or damaged organelles. The autophagic pathway basically comprises the sequestration of cytoplasmic materials in a double membrane vesicle called autophagosome and the later fusion of these vesicles with lysosomes to form autolysosomes. Simple compounds obtained after degradation of macromolecules are subsequently transported to cell cytosol and recycled as energy source substrates or biosynthetic precursors [1]. Autophagy is a cytoprotective process predominantly activated by stressful physiological stimuli such as starvation, oxidative stress or high levels of misfolded proteins contributing to cellular and tissue homeostasis [2]. Autophagy also participates in processes such as cell development and remodeling, programmed cell death, replacement of mitochondria and other organelles, elimination of aggregates of polyubiquitinated proteins and lipid metabolism [3]. Due to its participation as a quality control mechanism, autophagy is a higly regulated cellular process. Excessive or reduced autophagic activity contributes to the development of diseases such as cancer, neuronal disorders and myopathies, among others [4]. Autophagy has also been implicated in various aspects of innate and adaptive immunity [5,6], among which the capture and degradation of intracellular microorganisms has uncovered a specific antimicrobial role for autophagy.

The events that take place during autophagy are regulated by the so-called autophagy related genes (atg genes), which were initially described in yeast. So far, 32 genes involved in autophagy in mammals have been identified [7]. Their products, the ATG proteins, act sequentially to carry out the specific steps of autophagosome formation and maturation. The process starts with the formation of the isolation membrane or phagophore, which expands to entrap the cytoplasmic materials and finally closes to form the autophagosome [8]. Beclin-1, the mammalian homolog of the yeast Atg6, belongs to the VPS34 (vacuolar protein sorting 34) complex, a class III phosphatidylinositol 3-kinase (PI3K) complex, required for phagophore formation and enlargement [9–11]. Sequential association and dissociation of Atgs from the phagophore/autophagosomal membrane allows the maturation of autophagosomes in the autophagic pathway. LC3, the mammalian homolog of the yeast Atg8, is normally present as a cytosolic protein (LC3-I) in control conditions. However, upon autophagy induction, this protein conjugates with phosphatidylethanolamine to form LC3-II which associates to autophagic vesicles from the phagophore to the mature autolysosomes being the best marker for the study of autophagy [12,13]. Therefore, LC3 puncta formation detected by immunofluorescence or the increment of the LC3-II band, detected by immunoblotting analysis, reflects the existence of autophagosomes and allows monitoring autophagy. Recruitment and fusion of autophagosomes with late endosomes and lysosomes lead to the maturation of autophagic vacuoles in autolysosomes and the degradation of engulfed materials.

During the infectious processes, autophagy is, in addition to phagocytosis, a mechanism of the innate immune response (RII) that contributes to the elimination of intracellular pathogens [14], such as bacteria (group A streptococcus, *Mycobacterium tuberculosis, Salmonella enterica*, among others), protozoa (*Toxoplasma gondii*) and viruses (*Chikungunya* virus). In a process known as xenophagy [3], autophagosomes selectively capture free intracellular pathogens in the cytosol or inside phagosomes. These microorganisms, after transiting through the autophagic pathway, are killed by the lysosomal activity [15]. In the course of infection, autophagy can be stimulated by pathogen-associated molecular patterns (PAMPs) that are recognized by pattern recognition receptors (PRRs). The pathogen is then marked by ubiquitin, which, through the ubiquitin receptor p62 (SQSTM1), binds to LC3 and triggers the biogenesis of autophagosomes around the pathogen for its final degradation [16–18]. p62 is an adaptor protein that binds ubiquitinated cargo via its UBA domain and ATG8/LC3 via its LIR domain [19].

*Trypanosoma cruzi* is the causative agent of Chagas disease, also called American trypanosomiasis, which was firstly described by the Brazilian doctor, Carlos Chagas in 1909 [20]. The World Helth Organization estimates that there are almost 8 millon people infected with *T. cruzi* in at least 21 endemic countries of Latin America (https://www.who.int/chagas/epidemiology/en/) and the southern part of United States [21]. There are also a growing number of new cases in non-endemic countries such as European countries [22], Canada [23], New Zealand and Australia [24]. The disease has two clinical forms: an acute phase, followed by the chronic phase that can occur without an apparent pathology or clinical manifestations. In the acute phase, parasitemia levels are high and trypomastigotes, the infective form of the parasite, can be detected by microscopy in fresh blood preparations. Pericarditis can also be observed [25]. Parasitemia levels decrease around 90 days of infection. Approximately 30% of patients in the chronic phase develop cardiac symptoms due to the persistent presence of amastigotes, the intracellular replicative form of *T. cruzi* which form the so-called “amastigotes nests” in the myocardical fibers [26].

Using non-phagocytic cells, our laboratory previously showed that at early stages of *T. cruzi* infection, between 1 and 6 hours, trypomastigotes interact with autophagosomes visualized by the recruitment of LC3 protein to the *T. cruzi* parasitophorous vacuole (TcPV) at these times. The pre-induction of autophagy significantly increased the percentage of infected cells whereas this parameter was reduced in Atg5 KO MEFs and in HeLa cells transfected with small interfering RNA against Beclin-1, two situations which reduce the autophagic response of the host cell [27]. Induction of autophagy also increased the presence of late endosome/lysosome markers, such as LBPA, Lamp-1 or cathepsin-D in the TcPV indicating that the parasite exploits the autophagic pathway to increase the host cell infection by the lysosomal-dependent process [28]. Later after infection, when amastigotes were visualized in the cytoplasm, non-recruitment of autophagic proteins to parasites was observed [27]. Likewise, in human fibrosarcoma derived cells, Onisuka and coworkers found that LC3 did not colocalize with *T. cruzi* amastigotes. They also observed that autophagosomes were not matured in infected cells concluding that *T. cruzi* inhibits the autolysosome formation and the parasites were not enwrapped by autophagosomes in these cells [29].

The main objective of this work was to study the participation of autophagy in the development of *T. cruzi* infection in an *in vivo* model. We performed acute *T. cruzi* infections in *Beclin-1* heterozygous mutated mice (Bcln1^±^, Bcln KD), which displays a reduced autophagic response [30], comparing the course of the infection in the controls Bcln1^+/+^ (Bcln WT) mice. We also treated WT mice with autophagic inhibitors such as chloroquine [31,32] and difluoromethylornithine (DFMO) [33]. We found that autophagy-deficient mice developed higher parasitemia values and cardiac parasitic load with a reduced percentage of survival compared to control mice. We also analyzed the infection in peritoneal cells obtained from these mice, as well as RAW macrophages and confirmed the participation of autophagy in the reduction of *T. cruzi* intracellular infection. Further cellular studies with macrophages pointed to xenophagy as one of the mechanisms for elimination of intracellular parasites. We concluded that autophagy participates in the control of *T. cruzi* acute infection in mice.

## Results

### Establishment of a Trypanosoma cruzi acute infection model in mice.

To study the participation of autophagy during *T. cruzi* infection *in vivo*, we performed a previously standardized acute model of infection by using young (≈25 days old) C57BL/6 mice infected with tissue culture derived trypomastigotes (TCT) of *T. cruzi* Y-GFP strain [34]. At these conditions, *Beclin-1* wild type mice (Bcln^+/+^) developed high parasitemia values and lost weight significantly compared with non-infected mice (Figure S1(a)). These animals died between 14 and 21 days post-infection (dpi) as a consequence of the acute infection. In contrast, animals infected at the same conditions with *T. cruzi* K98, a previously described chronic strain of *T. cruzi* [35], died at later times from around 45 dpi to even 200 dpi (Figure S1(b)).

Previously published works have demonstrated the preferential reticular and muscle tissue tropism of *T. cruzi* Y strain in mice experimentally infected [36,37]. To confirm that our *T. cruzi* Y-GFP strain has a similar distribution to the Y wild-type strain and taking advantage that these parasites express GFP, we performed histology preparations of different tissues and organs from infected mice post-mortem and further analyzed by confocal microscopy. As shown in Figure S1(c), amastigote nests were clearly observed in many organs and tissues confirming the extensive distribution of the parasitism. Heart, liver, skeletal muscle, lungs and lymphoid organs showed moderate and high levels of nests abundancy whereas other tissues displayed low parasitism indicating that *T. cruzi* replication was likely not generated at these locations (Figure S1(d)). Overall, these data indicated that *T. cruzi* Y-GFP strain behaved similarly to the parental *T. cruzi* Y strain and exhibited comparable histopathology in mice.

### Autophagic deficiency increases the rate of T. cruzi infection in mice

**Figure 1.**
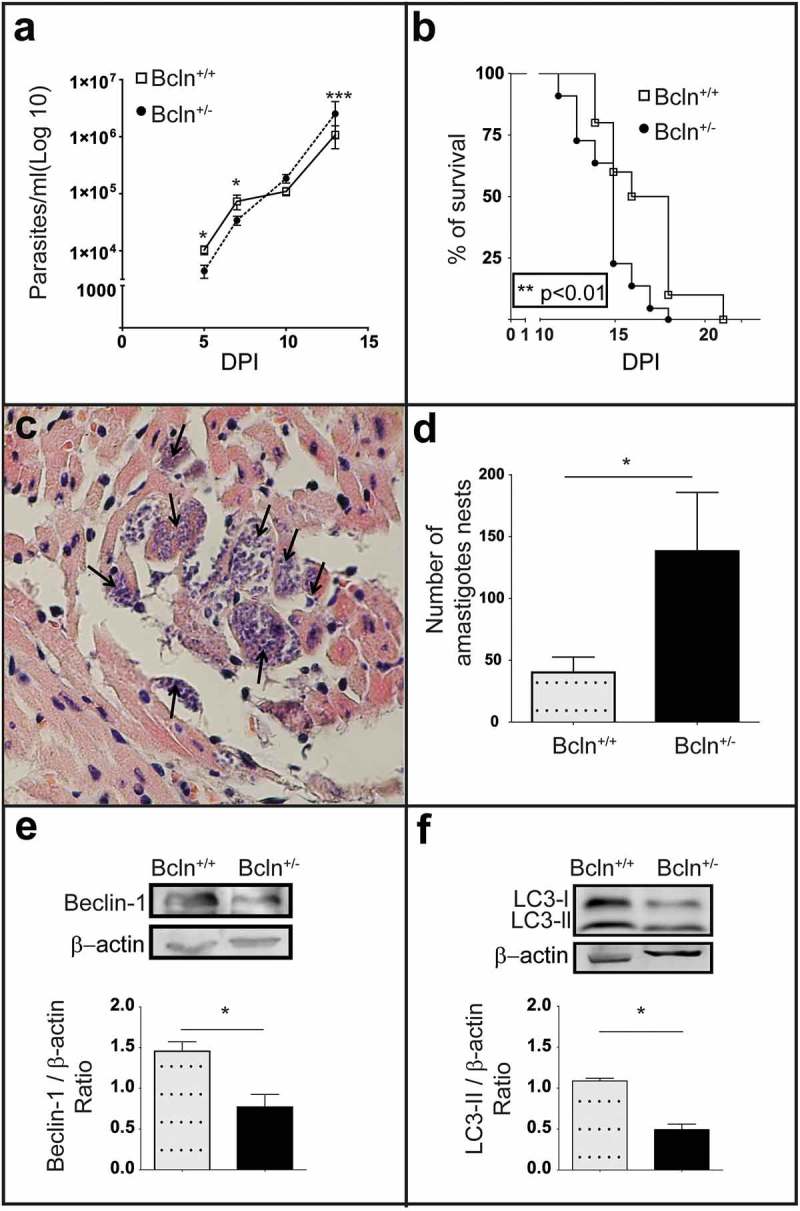
The deficiency of autophagy exacerbates *T. cruzi* infection in mice: (a): Parasitemia curve obtained from C57BL/6J wild type mice (Bcln^+/+^) and Beclin-1 heterozygous knock-out mice (Bcln^±^) infected with *T. cruzi* trypomastigotes of Y-GFP strain as described in materials and methods (Mann-Whitney test, * p < 0.05, *** p˂0.001). (b): Percentage of survival of Bcln^+/+^ and Bcln^±^ infected with *T. cruzi* Y-GFP. Data were obtained from three independent experiments of at least 4 mice each (log rank Mantel-Cox test, ** p˂0.01). (c): Cardiac histopathology by H&E staining. Arrows indicate nests of amastigotes. (d): Number of amastigotes nests in cardiac semi-serial sections. Data were obtained from two independent experiments of at least 4 mice each (Mann-Whitney test, * p < 0.05). (e): Representative image showing the expression of Beclin-1 in the liver of non-infected Bcln^+/+^ and Bcln^±^ mice by western blot. Graphic shows the OD of Beclin-1 band relative to β-Actin expression. Bars indicate the mean ± the standard error of two independent experiments with at least 2 mice each (Student´s t-test, * p < 0.05). F: Representative image showing the expression of LC3 forms in the liver of non-infected Bcln^+/+^ and Bcln^±^ mice by western blot. Graphic shows the OD of LC3-II relative to β-actin expression. Bars indicate the mean ± the standard error of two independent experiments of at least 2 mice each (Student´s t-test, * p < 0.05).

Next we performed the *T. cruzi* acute infection on Bcln^±^ mice and compared with Bcln^+/+^ animals. Since Beclin-1 is a member of the VPS34 complex required for autophagosome formation and a functional autophagy pathway [9,10,38], heterozygous disruption of the *Beclin-1* gene (*Bcln^±^*) results in a diminished autophagic response in mice [30,38]. Although many conditional knockout mice for Atg genes are available nowadays, up to date, only *Beclin-1* heterozygous mice express the mutation in the whole organism and are viable from the postnatal period up to adulthood [39], resulting in a good tool for our studies. The analysis of the *T. cruzi* infection course in the autophagy deficient mice showed significant differences when compared to wild type animals. At 5 and 7 dpi, parasitemia values were higher in Bcln^+/+^ animals than Bcln^±^. In contrast, at subsequent days (10 and 13 dpi), wild type mice displayed less parasitemia than Bcln^±^ which is evidenced by the almost constant values displayed by Bcln^+/+^ animals between 7 and 10 dpi (Figure 1(a)). On the other hand, parasitemia values in Bcln^±^ mice increased progressively from 5 to 13 dpi (Figure 1(a)). Of note, survival analysis showed that autophagy deficient animals displayed a significant earlier death (mean 14.68 SD = 0.3 days) compared to Bcln^+/+^ (mean 16.70 SD = 0.7 days) (Figure 1(b)). Parasitemia of Bcln^±^ animals at 10 dpi also correlated with the time of death (*r_S_ *= −0.66) suggesting that in these animals, the increased parasitemia values resulted in an earlier day of death. Furthermore, post-mortem analysis of myocardial tissues from infected mice, showed a significantly higher number of amastigote nests in Bcln^±^ mice compared to Bcln^+/+^ (Figure 1(c,d)). As expected, a lower level of Beclin-1 expression was detected in protein extracts obtained from livers of uninfected Bcln^±^ mice compared with Bcln^+/+^ (Figure 1(e)). Concomitantly, LC3-II detection was significantly reduced in the knock-down mice livers at control conditions, consistent with the reduced autophagic activity displayed by these animals (Figure 1(f)).

### Inhibition of autophagy enhances T. cruzi infection in wild type mice

**Figure 2.**
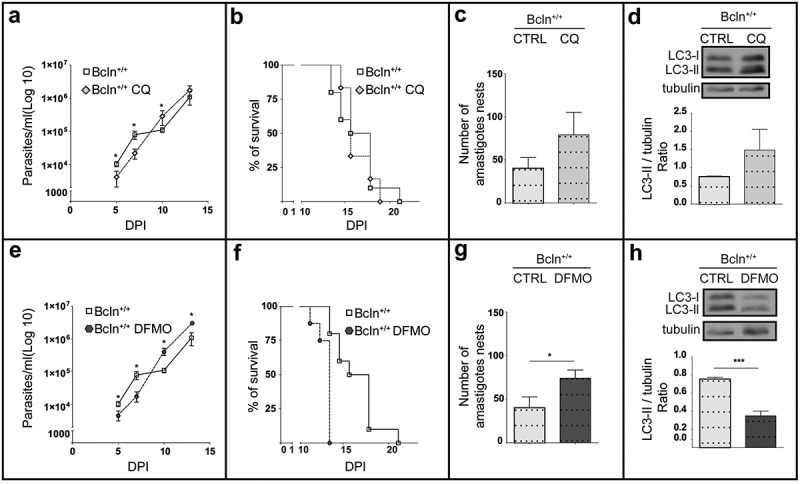
Inhibition of autophagy increases *T. cruzi* infection in Bcln^+/+^ mice. C57BL/6J wild type mice (Bcln^+/+^) were treated with CQ (10 mg/Kg/day) and DFMO (1 mg/g/day) for two days before infection and throughout the experiment as described in materials and methods. (a and e): Parasitemia curves obtained from these animals in comparison with non-treated Bcln^+/+^ mice. Data were obtained from three independent experiments of at least 4 mice each (Mann-Whitney test, * p < 0.05). (b and f): Survival analysis. Data were obtained from three independent experiments of at least 4 mice each (Log rank Mantel-Cox test) (c and g): Number of amastigotes in cardiac tissue from control and treated mice. Data were obtained from two independent experiments of at least 4 mice each (Mann-Whitney test, * p < 0.05). (d and h): Representative images showing the expression of LC3 forms in the liver of non-infected control or treated Bcln^+/+^ mice by western blot. Graphics show the OD of LC3-II relative to tubulin expression. Bars indicate the mean ± the standard error of two independent experiments of at least 2 mice each (Student´s t-test, *** p < 0.001).

To confirm the effect of the autophagic deficiency on *T. cruzi* infection in mice, we studied the outcome of the infection on the Bcln^+/+^ mice treated with two previously described autophagic inhibitors. Wild type mice were treated once a day with 10 mg/Kg/day chloroquine (CQ) or with 1 mg/g/day difluoromethylornithine (DFMO), from 2 days before infection until death (see details in Material and Methods). As mentioned above, the lysosomotrophic agent CQ is a potent autophagic flux inhibitor *in vivo* [31,32]. Additionally we tested the effect of DFMO, an inhibitor of the ornithine decarboxylase enzyme (ODC) that strongly reduces the intracellular level of spermidine, a polyamine that induces autophagy in several eukaryotic organisms [40]. This compound was previously described by our laboratory as a safe autophagic inhibitor which decreased the autophagic response *in vitro* by reducing the expression of Atg genes [33]. In agreement with our previous data, animals treated with DFMO showed a significant reduction in the total LC3 and Atg5 protein levels in livers when compared with untreated Bcln^+/+^ mice (Figure S2(a,b)). Additionaly DFMO treatment significantly reduced the amount of Beclin-1 (Figure S2(c)). Similar to Bcln KD mice, Bcln WT animals treated with the autophagic inhibitors reached higher parasitemia values at the end of the infection period (Figure 2(a,e)) and displayed an earlier death compared to the controls (Figure 2(b,f)). Cardiac amastigotes nests also increased in the treated mice (Figure 2(c,g)) demonstrating the higher level of infection displayed in conditions which inhibit autophagy. To control the autophagic response in treated mice the presence of LC3-II in livers normalized to tubulin was quantified. As expected CQ treatment increased the LC3-II/tubulin ratio by inhibition of autophagic flux whereas DFMO significantly decreased it, two opposite mechanisms which produced a similar effect of reduction of autophagy in treated mice (Figure 2(d,h)). Overall, these findings indicated that in contrast to the results obtained previously on cultured cells, the impairment of the autophagic activity in mice (by gene disruption or inhibitory compounds) enhanced the systemic infection of *T. cruzi* demonstrated by a higher parasitemia and cardiac load and an earlier death.

### Autophagy plays a role in the innate immune response against T. cruzi

**Figure 3.**
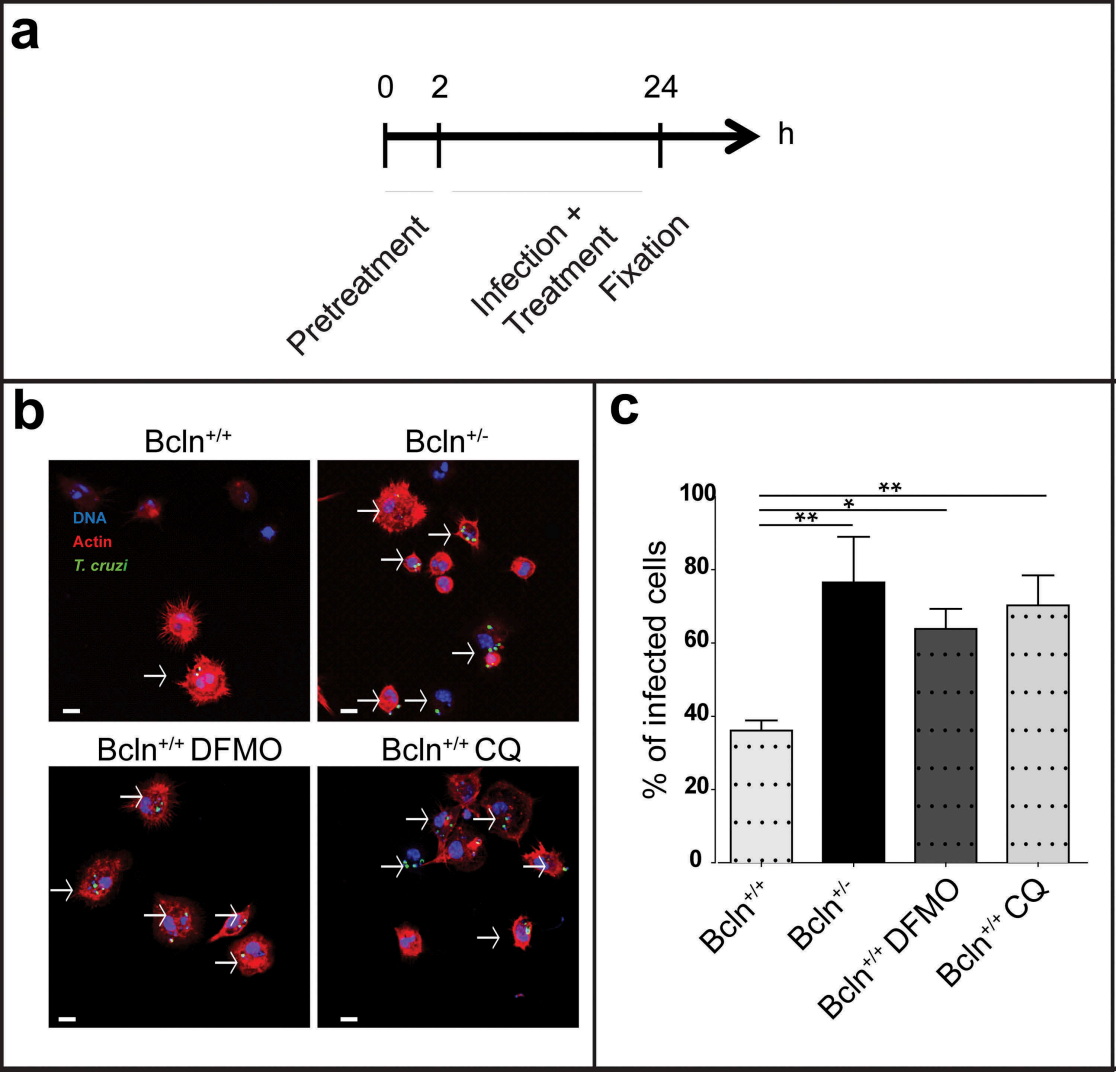
Inhibition of autophagy increases *T. cruzi* infection in peritoneal cells. Peritoneal cells were isolated by lavage of the peritoneal cavity of Bcln^+/+^ or Bcln^±^ mice and then infected with *T. cruzi* (MOI = 10) for 24 h before fixation. Samples, were then stained with TRITC-phalloidin and Hoechst and processed by confocal microscopy (see details in materials and methods). Other samples from Bcln^+/+^ mice were treated with 20 μM CQ or 10 mM DFMO from 2 h before and during infection and then processed as above. (a): Scheme of the infection protocol. (b): Representative images depicting the level of cellular infection under the indicated conditions. Parasites were visualized in green, actin cytoskeleton in red and the nuclei in blue. Bars: 5 µm. (c): Percentage of infected cells in the indicated conditions. Bars indicate the mean ± the standard error of at least three independent experiments. Number of counted cells: 100 to 300 cells each experiment (Dunnet test, * p < 0.05; ** p < 0.01).

Given that trypomastigotes were inoculated to animals by IP injection, we next characterized the infection in peritoneal cells (PC), the primary focus of infection in our system. As explained in Materials and Methods, these cells were obtained by a careful washing of the peritoneal cavity of healthy Bcln^+/+^ and Bcln^±^ animals, plated and then infected *in vitro* during 24 h before fixation. Other samples of PC from Bcln^+/+^ mice were treated in the presence of 1 mM DFMO or 20 μM CQ from 2 h before infection until 24 h (Figure 3(a)). After fixation all samples were processed by confocal microscopy analysis. PC cytoplasm and nucleus were labeled with TRITC-phalloidin and Hoechst respectively, whereas intracellular parasites were directly visualized in green due to the stable expression of *TcH2b* histone fused to GFP [41] (Figure 3(b)). Interestingly, PC obtained from Bcln^±^ or from Bcln^+/+^ and treated with inhibitors displayed significantly higher infection than cells obtained from Bcln^+/+^ animals maintained in control conditions (Figure 3(b,c)), indicating that a reduced autophagic activity was related to higher infection in peritoneal cells. In agreement with the results obtained with animals, experiments with peritoneal cells showed that autophagy is a process which participates in the control of *T. cruzi* infection.

**Figure 4.**
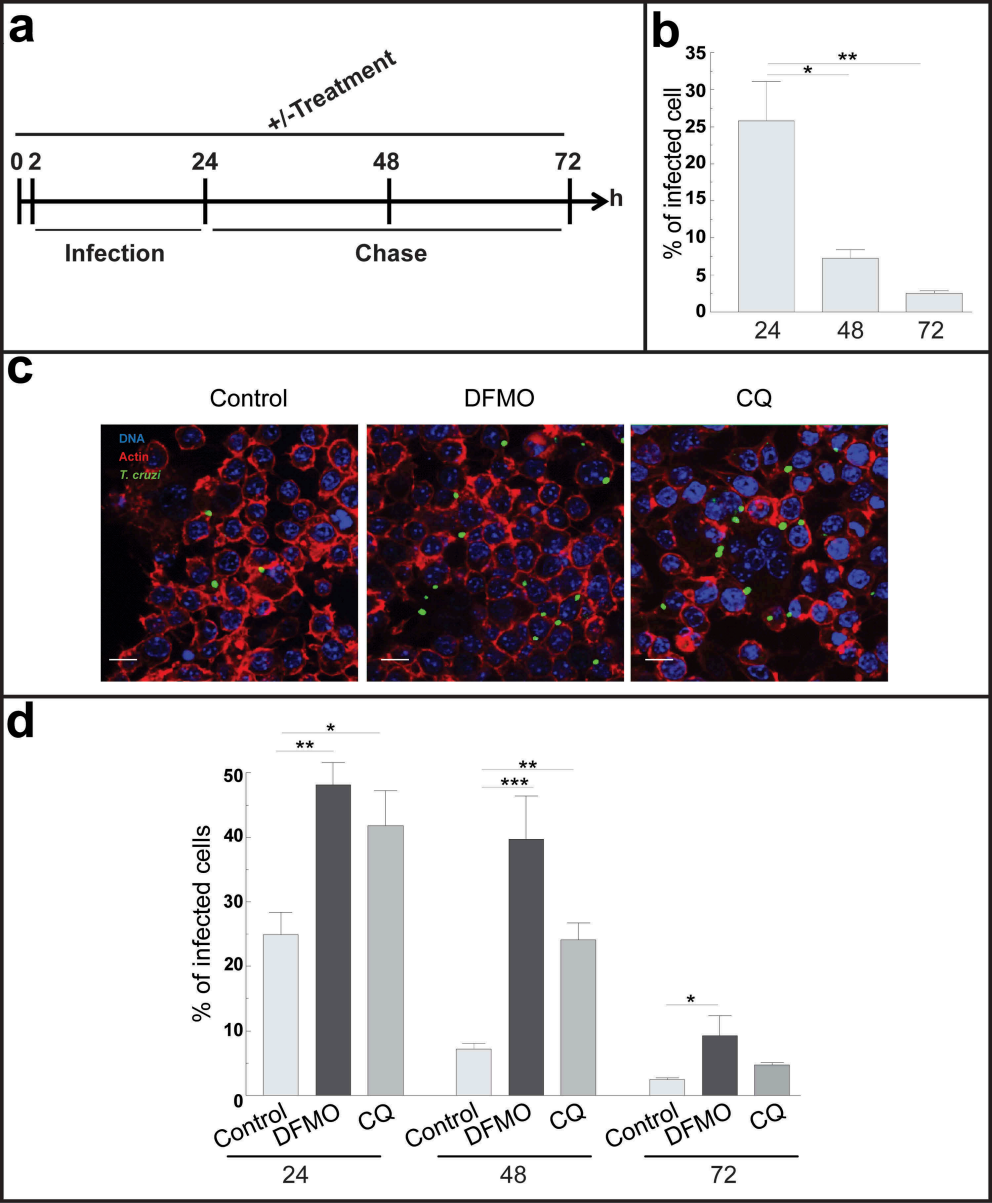
Autophagy participates in the control of *T. cruzi* infection by macrophages. RAW macrophages were infected for 24 h, washed and fixed or incubated for 24 or 48 h more before fixation. Fixed samples were then processed for microscopy (see details in materials and methods). Other samples were treated with 10 mM DFMO or 20 μM CQ for 2 h before infection (pre-treatment) and maintained in the same conditions during infection and chase periods followed by fixation and processing. (a): Scheme of the protocol. (b): Percentage of macrophages infected at progressive times in control conditions. Bars indicate the mean ± the standard error of at least four independent experiments. Number of counted cells: 100 (Tukey test, *p < 0.05; **p < 0.01). (c): Representative images depicting the level of cellular infection at 24 h under the indicated conditions. Parasites were visualized in green, actin cytoskeleton in red and the nuclei in blue. Bars: 5 μm. (d): Percentage of infected cells in the absence (control) or the presence of the autophagic inhibitors DFMO or CQ at each indicated time. Bars indicate the mean ± the standard error of at least three independent experiments. Number of counted cells: 100 to 300 cells each experiment (Tukey test, * p < 0.05; **p < 0.01; ***p < 0.001).

In our experimental model, innate immunity is the main component of the immune response since mice develop an acute infection and die at very early times after infection. Several reports in the literature have demonstrated that macrophages are the first line of defense against *T. cruzi* suppressing parasite replication and spreading in host tissues during the acute phase of infection [42]. Furthermore, 30% of peritoneal cells are naïve resident macrophages [43] indicating that one of the first targets of *T. cruzi* in our model were peritoneal macrophages. Taken these considerations, we next analyzed the course of *T. cruzi* infection in macrophages by performing kinetic experiments in RAW cells, a murine macrophage cell line. Cells were infected with TCT from the Y-GFP strain during 24 h. After this period of time, extracellular parasites were removed by washing and one coverslip, corresponding to the first time point (24 h), was immediately fixed. The other samples were incubated in control medium for an additional period to complete a total assay time of 48 and 72 h before fixation. In another set of experiments, cells were incubated with the autophagy inhibitors DFMO or CQ for 2 h and maintained in the same conditions during the infection and chase (± treatment) before fixation (Figure 4(a)). After fixation, samples were processed and analyzed by confocal microscopy (see Materials and Methods). Interestingly, in control conditions (untreated cells) we observed a progressive reduction in the infection from early (24 h) to later times (72 h) after infection, whereas the number of macrophages was almost not modified at these times. Quantification studies showed a significant decrease in the percentage of infected cells from 24 to 48 and 72 h after infection (Figure 4(b)). In contrast, in the presence of autophagy inhibitors we observed a bigger infection compared to control (Figure 4(c)). Quantitative data confirmed a significant increase in the percentage of infection under DFMO and CQ treatment compared to cells under control conditions at each time (Figure 4(d)). Taken together these data indicated that macrophages are able to clear the intracellular parasites through the course of the infection, autophagy being one of the mechanisms involved in this elimination.

**Figure 5.**
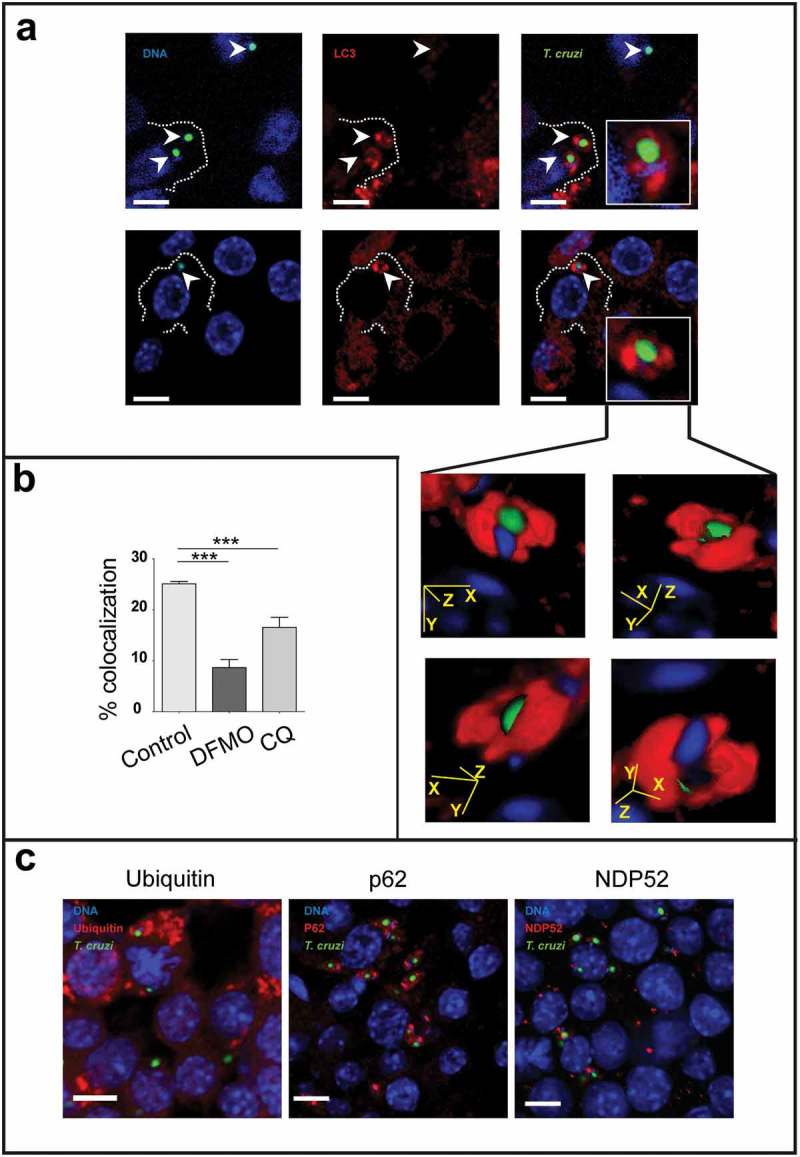
Macrophages eliminate intracellular parasites by xenophagy. RAW macrophages were infected for 24 h, washed and incubated for an additional period of 24 h in control medium or in the presence of 20 μM CQ or 1 mM DFMO before fixation. Fixed cells were then processed to detect LC3, ubiquitin, p62 or NDP52 by indirect immunofluorescence (see details in materials and methods). (a): Images depicting intracellular parasites (visualized in green and blue) surrounded by LC3 protein (in red). Insets show LC3 position of four different stacks of an amastigote. Bars: 5 µm. (b): Percentage of parasites surrounded by LC3 at different conditions. Bars indicate the mean ± the standard error of at least three independent experiments. Number of counted cells: 100 to 300 cells each experiment (Dunnet test, ***p < 0.001). (c): Images depicting different autophagy/xenophagy markers (in red) recruited to *T. cruzi* amastigotes (in green and blue).

After invasion of cells, trypomastigotes transiently reside in a vacuole which membrane is then degraded to release the parasites to the cytoplasm. Cytosolic trypomastigotes differentiate into amastigotes which then initiate their multiplication. At this point we were interested in determining whether *T. cruzi* amastigotes could be captured by autophagy in the cytoplasm of infected macrophages. To this end we conducted experiments similar to the one described above. RAW cells were infected for 24 h and, after removal of non-internalized parasites; cells were incubated for an additional time of 24 h to allow the progression of the *T. cruzi* intracellular cycle, in the presence or not of the autophagic inhibitors. After fixation, cells were processed to detect the LC3 protein by indirect immunofluorescence, and analyzed by confocal microscopy. As shown in Figure 5(a), we found *T. cruzi* amastigotes (labeled in green and blue) decorated with LC3 (labeled in red). This recruitment was observed in aproximately 25% of the parasites when cells were maintained without inhibitors but significantly decreased under DFMO and CQ treatment indicating that a functional autophagy is required for the association of LC3 to amastigotes (Figure 5(b)). As mentioned in the introduction, LC3 positive vesicle engulfment of a pathogen and the further elimination of it through the autophagic pathway is known as xenophagy [16–18]. Therefore, the possible recruitment to amastigotes of other proteins related to xenophagy was determined. Besides LC3, we found that p62 and NDP52, two adaptors previously described in the binding of LC3 to pathogens, were highly recruited at 48 h. At earlier times (24 h) we also found ubiquitin, the first molecule that recognizes and binds to pathogens (Figure 5(c)). Taken together, these data indicated that, by engagement of xenophagy, autophagy participates in the elimination of *T. cruzi* in macrophages.

## Discussion

Autophagy is a degradative pathway involved in several homeostatic processes. As detailed in the introduction, an important body of evidences indicates that autophagy is also a component of the immune system, being involved in both innate and adaptive responses [44]. *T. cruzi* is the parasite that causes Chagas disease, one of the most widely spread infective illnesses in the world which impairs the health of wide populations and generates enormous economic costs. Previous *in vitro* studies from our laboratory demonstrated that autophagy is a key element during infection caused by *T. cruzi* in non-phagocytic cells. By live-cell microscopy we showed the recruitment of LC3 positive vesicles to the sites of entry of *T. cruzi*. LC3 protein was also observed at the membrane of the TcPV [27]. Induction of autophagy previous to infection enhanced parasite colonization by increasing the number of lysosomes which, in turn, leads to the internalization and the maturation of the parasitophorous vacuole, an essential process to retain the infection inside host cells [28]. Later studies also demonstrated that inhibition of autophagy by treatment with DFMO impaired *T. cruzi* infection [33]. Considering these previous studies, we have speculated that the impairment of the normal autophagic response, during the infection with *T. cruzi* in a mouse model, would benefit the host by a reduction of the capacity of parasites to internalize and maintain inside host cells. Surprisingly, mice deficient in autophagy underwent a more aggressive infection, displaying higher values of parasitemia and cardiac parasitism that lead to an earlier death. In agreement with these results, a previous study showed that *M. tuberculosis* infection of Atg5 conditional knock out mice resulted in increased bacillary burden and excessive pulmonary inflammation compared to autophagy-proficient littermates [45].

Interestingly, the curve of parasitemia of WT animals can be divided in three periods; at early times after infection (5 and 7 dpi) has a high slope and reaches higher values, statistically significant, than Beclin-1 KD mice or WT animals treated with the autophagic inhibitors, CQ and DFMO. In the second period, between 7 and 10 dpi, the curve is flattened (low slope) and later, between 10 and 13 dpi, climbed again with a slope similar to the first period. In contrast, autophagy deficient mice exhibited a progressively increased curve with a similar slope for the whole studied period. The strikingly constant parasitemia values (at 7 and 10 dpi) of Beclin^+/+^ mice suggested the presence of an active antiparasitic response of these animals that was not observed in Beclin^±^ mice or in the presence of inhibitors of autophagy which displayed a constant increment of the values of parasitemia accordingly to the progress of the infection. Unexpectedly, WT animals displayed higher values of parasitemia trough the first stage of infection until 7 dpi, indicating that, although in experiments with isolated peritoneal cells from Beclin-1 KD mice showed an increased infection compared to WT, some delay is produced perhaps in the exit of parasites from the cells in the first infective cycle, or alternatively, in the transit required to reach the bloodstream in KD animals. Indeed, it has been demonstrated that the macrophage migration inhibitory factor MIF, a cytokine previously described as a component of the primary response against *T. cruzi* [46], triggered autophagic degradation of junction proteins and increased the permeability of human endothelial cells [47]. It is possible that KD mice displayed an impaired response to MIF and vascular permeability is not increased as efficiently as in WT animals resulting in a minor (or delayed) exit of parasites to the bloodstream. More experiments will be necessary in the future to confirm this hypothesis. Independently of these initiall differences, all animals finally die due to the acute infection; however, WT animals died at later times displaying a greater ability to resist the infection.

To disclose the possible mechanisms of this resistance to infection, we focus our study in the function of macrophages. These cells are the first line of defense against *T. cruzi* in acute infections [42] and are also present in the peritoneal cavity, the site of initial infection. In agreement with the animal parameters, peritoneal cells from WT animals showed a lower infection than both Beclin^±^cells and WT cells treated with DFMO or CQ. Infection of RAW cells was also significantly decreased when compared early to later times, which highlight their ability to fight *T. cruzi*. In contrast, inhibition of autophagy impaired this antiparasitic activity evidenced by the increased infection measured in these cells subjected to DFMO or CQ treatment.

It has been described that autophagy participates in the innate immune responses by sequestering intracellular pathogens to further degradation inside autolysosomes [16]. This process, so-called xenophagy, was described for both intraphagosomal bacteria and cytoplasmic-living pathogens. Although *M. tuberculosis* produced a phagosomal maturation arrest to avoid being killed by lysosomal degradation [48], it was demonstrated that induction of autophagy on infected macrophages suppressed intracellular survival of mycobacteria in a process dependent on the recruitment of LC3 to the membrane of mycobacterial phagosomes and the further colocalization with lytic compartments [49]. In a similar way, pathogenic group A Streptococcus were enveloped by autophagosome-like compartments and killed upon subsequent fusion of these compartments with lysosomes [50]. After these seminal studies, many other intracellular pathogens were found to be tagged for autophagic degradation demonstrating the crucial role of xenophagy in the innate immune response [51].

Interestingly we found the LC3 protein surrounding amastigotes formed at later times of infection, from 24 h towards, suggesting that xenophagy is participating in the control of *T. cruzi* infection by macrophages. We also found ubiquitin and the autophagic receptors p62 and NDP52, all components of autophagic selective processes [52]. *Toxoplasma gondi*, another protozoan intracellular pathogen was also enveloped by LC3 and targeted by autophagic degradation in macrophages activated by the toxoplasmocidal CD40 ligand [53]. We conclude that xenophagy is involved in the elimination of parasites by macrophages and that absence of autophagy impairs this process resulting in an increased infection both in cells and organisms. In addition, other trypanocidal mechanisms could be also affected by the absence of autophagy. It is known that oxidative metabolism activated in macrophages after *T. cruzi* infection drives nitric oxide generation and pathogen clearance [54]. As one of the main regulators of cellular metabolism, autophagy may also be participating in these oxidative responses of macrophages.

It is interesting to note that the fate of *T. cruzi* infection in a cell can be different according to the class of host cell infected. In contrast to phagocytic cells that activate an immune response that tend to control the infection [42]; in non-phagocytic cells, *T. cruzi* can survive, replicate and transmit the infection to other cells. Concerning to autophagy in this context, Onisuka and co-workers found in a non-phagocytic cell line that although *T. cruzi* infection produced an increased number of autophagosomes in the cells, amastigotes were not enwrapped by them, suggesting that the parasite has mechanisms that allows it to evade the autophagic capture [29]. In agreement with these results, previous data from our lab showed, in epithelial cells, an increment of LC3-positive vesicles after *T. cruzi* infection [55] and that with the exception of few cases, LC3 is not recruited to amastigotes at later times of infection [27]. Eventhough, a different scenario was observed in macrophages. In these phagocytic cells we observed that around 25% of amastigotes were surrounded with LC3 and also with some molecular components of the xenophagy machinery such as ubiquitin and the adaptors p62 and NDP52. Taken together, these findings indicate that macrophages at least partially, can overcome the mechanisms of evasion of autophagy displayed by *T. cruzi*. Therefore, the action of autophagy against *T. cruzi* in macrophages can explain in part the major resistence of WT animals to infection when compared to autophagic-deficient mice. We concluded that, in contrast to what occurs in non-phagocytic cells *in vitro*, autophagy plays a protective role against the infection caused by *T. cruzi* in mice, being involved in the repertoire of immune responses generated by the host. Due to chronic Chagas disease pathology involves the persistence of parasites on specific tissues (mainly heart) and also the generated immune response that is responsible of damaging; all efforts to understand the mechanisms which participate in the immunity against this parasite will benefit the finding of new strategies to combat this illness in the future.

## Materials and methods

### Reagents

Minimum essential medium (α-MEM) and Dulbecco modified minimal essential medium (D-MEM) was obtained from Gibco Laboratories (Buenos Aires, Argentina). Fetal bovine serum (FBS) was purchased from Natocor S.A. (Córdoda, Argentina). The inhibitors α-difluoromethylornithine (DFMO) and Chloroquine (CQ) and the TRITC-conjugated phalloidin was purchased from Sigma (Buenos Aires, Argentina). The rabbit anti-Beclin-1 antibody was purchased from Santa Cruz (Santa Cruz Biotechnology, INC) and the polyclonal rabbit anti-LC3 antibody from Sigma (Buenos Aires, Argentina). The following antibodies were also used: anti-Atg5 (Abcam), anti-p62 (Abcam), Anti-ubiquitin (Santa Cruz), anti-β-actin (Genescript) and the anti-β tubulin (E7) was obtained from Developmental Studies Hybridoma Bank. The secondary antibodies Cy3-conjugated anti-mouse and Cy3-conjugated anti-rabbit were purchased from Jackson Immuno Research Laboratories, INC, as well as the antibodies HRP-conjugated anti-rabbit IgG, HRP-conjugated anti-mouse IgG and HRP-conjugated Anti-goat IgG. Hybond-ECL nitrocellulose membranes were from Amersham. The DNA marker Hoechst 33342 was purchased from Life Technologies.

### Parasites

Trypomastigotes of the K98 chronic strain [35] were donated by Dr. Stella Maris González Cappa (Department of Microbiology, Faculty of Medicine, University of Buenos Aires, Buenos Aires, Argentina). For the acute infections, we used trypomastigotes from the Y-GFP strain. This strain, which express the H2b histone fused to GFP [41], were generously provided by Dr S. Schenkman (Department of Microbiology, Immunology and Parasitology, Federal University of Sao Paulo, Brazil). Y-GFP metacyclic trypomastigotes were obtained from the Y-GFP epimastigotes by *in vitro* metaciclogenesis following the method described in Contreras and Figueiredo [56,57]. Metacyclic trypomastigotes were then used to infect Vero cells from which, TCT were further generated. TCT from the third replication cycle in Vero cells were used for animal infection.

### Mice

We used C57BL/6J wild-type mice (WT, Bcln^+/+^) and mice heterozygous knock-out for the gene Beclin-1 (Bcln KD, Bcln^±^) [30] of both sex, ≈25 days old, with an initial weight between 9–12 g [58]. These mice were generously provided by Dr. Beth Levin (Department of Microbiology, University of Texas Southwestern Medical Center, Dallas, TX, USA). The animals were kept in conditions free of specific pathogens (SPF) and housed in cages with controlled temperature and humidity. They were given water and food *ad libitum*.

### Infeccion

The animals were anesthetized with a medium-duration dose of 50 mg/kg Ketamine and 10 mg/kg Xylazine, administered intraperitoneally (IP). Then they were injected by the same route with 100,000 blood trypomastigotes (BT) of *T. cruzi* K98 strain or with 100,000 TCT of *T. cruzi* Y-GFP strain.

### Treatment

The treatment was started 2 days before infection and continued until the day of the animal death. It was administrated with a syringe into the intraperitoneal cavity. The CQ was dispensed daily in a dose of 10 mg/Kg/day for each animal, while DFMO was administered daily in a dose of 1 mg/g/day. The control group was treated with 200 μl of saline solution at a concentration of 9 g/L.

### Evolution of infection

Parasitemia was evaluated by counting the parasite number present in peripheral blood taken from the tail by direct puncture. At 5 and 7 dpi, mycroscopic examination was carried out by under cover slips (24mm x 24 mm), by counting the parasite number in 10 fields (400x) [59]. At 10 and 13 dpi parasitemia was assessed by counting the parasites in a Neubauer chamber [60] and the results were expressed in parasites/ml. In the same time periods weights were evaluated for each subject.

### Ethics

All animal procedures were cared in accordance with the Guiding Principles in the Care and Use of Animals of the US National Institute of Health. All procedures were approved by the Institutional Animal Care and Use Committee of the School od Medical Science, Universidad Nacional de Cuyo (Protocol approval N° 107/2017).

### Histopathology

After the death, the following organs were removed: heart, liver, spleen, thymus, skeletal muscle (quadriceps), lung, kidney and fat. These organs were subsequently introduced into a vial containing a buffered solution of 10% saline formaldehyde to be fixed before processing. Tissues were then embedded in paraffin, cutted at 5 µm and stained with Hoechst. Samples were examined by confocal microscopy using an Olympus Confocal FV1000 (Japan) and processed with the program FV10-ASW 1.7.

For the quantification of cardiac amastigotes nests, six semi-serial sections with 70-μm intervals, were obtained for each heart and stained with hematoxylin-eosin [61]. Twenty five (25) slices per field were analyzed by conventional microscopy using an Olympus CX31 microscope.

### Cell culture

RAW 264.7 cells were grown on in D-MEM supplemented with 10% FBS and antibiotics at 37ºC in an atmosphere of 95% air and 5% CO_2_. Cells were plated on coverslips in 24 well-plates to 80% confluence before experiments.

### Peritoneal Cells (PCs) isolation

PCs were obtained by peritoneal lavage of euthanized animals (by sedation with Ketamine/Xylazine followed by cervical dislocation) from uninfected mice. Cells were harvested from the peritoneal cavity by injecting cold PBS, centrifuged at 500 g for 5 min at 4°C, and resuspended in D-MEM complete medium supplemented with 10% FBS and antibiotics to a final concentration of 1 × 10^6^ cells/ml. The PC suspension was dispensed into 24-well flat-bottom plates and incubated at 37°C, 5% CO_2_, for 24 h, to allow the cells, mainly macrophages, to adhere to the plastic surface, followed by vigorous washing with PBS to remove nonadherent cells.

### Microscopy and indirect inmunofluorescence

RAW cells or PCs were plated (50.000 and 25,000 cells/well respectively) and incubated in control medium (D-MEM) in the presence or absence of autophagy inhibitors, 1 mM DFMO [33] or 20 µM CQ [62]. The infection was carried out with *T. cruzi* Y-GFP strain in a 10: 1 ratio. In all cases cells were fixed with a 3% solution of paraformaldehyde in PBS for 15 min at room temperature. After washing with PBS, cells were blocked for 15 min with 50 mM NH_4_Cl in PBS, permeabilized with a 0.2% solution of albumin and 0.1% saponin in PBS. After permeabilization, LC3, Ubiquitin, p62 or NDP52 proteins were detected by indirect immunofluorescence by using specific antibodies followed by detection with Cy3 tagged secondaries antibodies before mounting with Mowiol containing Hoeschst and examined by confocal microscopy in a confocal Olympus FV1000 microscope. Alternatively, RAW cells were stained with rhodamine-phalloidin to facilitate the quantification of the percentage of infected cells.

### Autophagy inhibition and infection

RAW or PC grown in control medium were washed three times with PBS and plated in 24 well-plates. In some experiments cells were incubated in control medium or in the presence of the drugs for 2 h prior to infection with *T. cruzi* Y-GFP (10:1 parasite:cell ratio) and continued for additional time of 24 h. In other cases, the cells were infected during 24 h and, after washing, treated with drugs for an additional time of 24 h or incubated in control medium during 24 or 48 h before fixation. Cells were then fixed with 3% paraformaldehyde solution in PBS for 15 min at room temperature, washed with PBS, and blocked with 50 mM NH_4_Cl in PBS. After washing, cells were mounted with Mowiol containing Hoechst and examined by confocal microscopy, using an Olympus Confocal FV1000 (Japan). Images were processed with the program FV10-ASW 1.7. Confocal images of 10 random fields were quantified, representing around 100 cells per experiment. Data are presented as mean values and error bars indicate the SEM from at least three independent experiments.

### Western blot

Liver protein extracts from healthy mice were used for western blot studies. The tissue was lysed in a homogenization buffer (20mM M Tris-HCl pH 8, 250mM saccharose, 0.1M EDTA pH 8.5 and 0.01% PMSF on ice. Samples were then centrifuged at 1000 × g for 10 minutes at room temperature, and the supernatants were used as protein extracts. Equal amounts of proteins were resolved in 12.5% (for LC3, Beclin-1 and Atg5) SDS-polyacrylamide gel electrophoresis and transferred to Hybond-ECL (Amersham) nitrocellulose membranes. Afterwards they were incubated in blocking solution (5% non-fat milk, 0.05% Tween 80 and PBS) for 1 h at room temperature, washed four times in PBS-Tween 80 (PBS, 0.05% Tween-80) and incubated with the corresponding primary antibodies (anti-LC3, anti-Beclin-1 or anti-Atg5), overnight at 4°C. The membranes were then washed three times for 10 min each in PBS-Tween 80 and incubated with peroxidase-conjugated secondary antibodies (Jackson Immuno Research) for 2 hours at room temperature. After three washes with PBS-Tween 80, the membrane-bound antibody was visualized by an enhanced chemiluminescence detection kit from Healthcare (Amersham, RPN2109) and the band was detected using Fujifilm LAS-4000 equipment.

### Statistic analysis

The data is presented as the mean ± the standard error. Student’s t test or ANOVA followed by Dunnet’s multiple comparisons test were used for parametric analyzes whereas Mann-Whitney test or Kruskal-Wallis test followed by the Dunn multiple comparisons test were used in nonparametric tests. The analysis of mortality was done by the log-rank [Mantel-Cox] test. In all cases the analyzes were carried out using Prism (GraphPad software, V. 5.0). The significance corresponds to * p < 0.05; ** p < 0.01 and *** p < 0.001.

## References

[1] Loos B, Engelbrecht A-M, Lockshin RA, et al The variability of autophagy and cell death susceptibility. Autophagy. [Internet]. 2013 [cited 2018 828];9:1270–1285. Available from: http://www.ncbi.nlm.nih.gov/pubmed/23846383 23846383 10.4161/auto.25560 PMC4026026

[2] Vacek TP, Vacek JC, Tyagi N, et al Autophagy and heart failure: a possible role for homocysteine. Cell Biochem Biophys. 2012;62:1–11. 21910028 10.1007/s12013-011-9281-6

[3] Choi AMK, Ryter SW, Levine B. Autophagy in human health and disease. N Engl J Med. 2013;368:1845–1846. 10.1056/NEJMc1303158 23656658

[4] Levine B, Kroemer G Autophagy in the pathogenesis of disease. Cell. 2008;132:27–42. 18191218 10.1016/j.cell.2007.12.018 PMC2696814

[5] Levine B, Mizushima N, Virgin HW Autophagy in immunity and inflammation. Nature. 2011;469:323–335. 21248839 10.1038/nature09782 PMC3131688

[6] Deretic V Multiple regulatory and effector roles of autophagy in immunity. Curr Opin Immunol. 2009;21:53–62. 19269148 10.1016/j.coi.2009.02.002 PMC2788943

[7] Pyo JO, Nah J, Jung YK Molecules and their functions in autophagy. Exp Mol Med. 2012;44:73–80. 22257882 10.3858/emm.2012.44.2.029 PMC3296815

[8] Tooze SA, Dooley HC, Jefferies HBJ, et al Assessing mammalian autophagy. Methods Mol Biol. 2015;1270:155–165. 10.1007/978-1-4939-2309-0_12 25702116

[9] Aita VM, Liang XH, Murty VV, et al Cloning and genomic organization of beclin 1, a candidate tumor suppressor gene on chromosome 17q21. Genomics. 1999;59:59–65. 10395800 10.1006/geno.1999.5851

[10] Kihara A, Kabeya Y, Ohsumi Y, et al Beclin-phosphatidylinositol 3-kinase complex functions at the trans -Golgi network. EMBO Rep. 2001;2:330–335. 11306555 10.1093/embo-reports/kve061 PMC1083858

[11] Zhong Y, Wang QJ, Li X, et al Distinct regulation of autophagic activity by Atg14L and Rubicon associated with Beclin 1–phosphatidylinositol-3-kinase complex. Nat Cell Biol. 2009;11:468–476. 19270693 10.1038/ncb1854 PMC2664389

[12] Walczak M, Martens S Dissecting the role of the Atg12-Atg5-Atg16 complex during autophagosome formation. Autophagy. 2013;9:424–425. 23321721 10.4161/auto.22931 PMC3590266

[13] Kabeya Y, Mizushima N, Ueno T, et al LC3, a mammalian homologue of yeast Apg8p, is localized in autophagosome membranes after processing. Embo J. 2000;19:5720–5728. 11060023 10.1093/emboj/19.21.5720 PMC305793

[14] Yu X, Li C, Hong W, et al Autophagy during Mycobacterium tuberculosis infection and implications for future tuberculosis medications. Cell Signal. 2013;25:1272–1278. 23416463 10.1016/j.cellsig.2013.02.011

[15] Deretic V, Levine B Autophagy, immunity, and microbial adaptations. Cell Host Microbe. 2009;5:527–549. 19527881 10.1016/j.chom.2009.05.016 PMC2720763

[16] Knodler LA, Celli J Eating the strangers within: host control of intracellular bacteria via xenophagy. Cell Microbiol. 2011;13:1319–1327. 21740500 10.1111/j.1462-5822.2011.01632.x PMC3158265

[17] Ma Y, Galluzzi L, Zitvogel L, et al Autophagy and cellular immune responses. Immunity. [Internet]. 2013 [cited 2018 829];39:211–227. Available from: http://www.ncbi.nlm.nih.gov/pubmed/23973220 23973220 10.1016/j.immuni.2013.07.017

[18] Weil R Does antigen masking by ubiquitin chains protect from the development of autoimmune diseases? Front Immunol. 2014;5. 10.3389/fimmu.2014.00262 PMC4042494 24917867

[19] Randow F How cells deploy ubiquitin and autophagy to defend their cytosol from bacterial invasion. Autophagy. 2011;7:304–309. 21193841 10.4161/auto.7.3.14539

[20] Romana C [The developmental cycle of Trypanosoma (Schizotrypanum) cruzi Chagas 1909. in its tissular and hematic phases]. Memorias do Instituto Oswaldo Cruz. 1956;54:255–269. 13369154 10.1590/s0074-02761956000100010

[21] Bern C, Kjos S, Yabsley MJ, et al Trypanosoma cruzi and Chagas’ disease in the United States. Clin Microbiol Rev. 2011;24:655–681. 21976603 10.1128/CMR.00005-11 PMC3194829

[22] Perez-Molina JA, Perez-Ayala A, Parola P, et al EuroTravNet: imported Chagas disease in nine European countries, 2008 to 2009. Euro Surveill. 2011;16:pii: 19966. 21944557

[23] Schipper H, McClarty BM, McRuer KE, et al Tropical diseases encountered in Canada: 1. Chagas’ disease. Can Med Assoc J. 1980;122:165–169, 171–172. PMC1801746 6767543

[24] Pinto A, Pett S, Jackson Y Identifying Chagas disease in Australia: an emerging challenge for general practitioners. Aust Fam Physician. 2014;43:440–442. 25006603

[25] Corrêa VR, Barbosa FG, de Melo CA Junior, et al Uneventful benznidazole treatment of acute Chagas disease during pregnancy: a case report. Rev Soc Bras Med Trop. 2014;47:397–400. 25075496 10.1590/0037-8682-0250-2013

[26] Chatelain E, Konar N Translational challenges of animal models in Chagas disease drug development: a review. Drug Des Devel Ther. 2015;9:4807. 10.2147/DDDT.S90208 PMC4548737 26316715

[27] Romano PS, Arboit MA, Vázquez CL, et al The autophagic pathway is a key component in the lysosomal dependent entry of Trypanosoma cruzi into the host cell. Autophagy. 2009;5:6–18. 19115481 10.4161/auto.5.1.7160

[28] Andrade LO, Andrews NW The Trypanosoma cruzi–host-cell interplay: location, invasion, retention. Nature Rev Microbiol. 2005;3:819–823. 16175174 10.1038/nrmicro1249

[29] Onizuka Y, Takahashi C, Uematsu A, et al Inhibition of autolysosome formation in host autophagy by Trypanosoma cruzi infection. Acta Trop. 2017;170:57–62. 28232068 10.1016/j.actatropica.2017.02.021

[30] Qu X, Yu J, Bhagat G, et al Promotion of tumorigenesis by heterozygous disruption of the beclin 1 autophagy gene. J Clin Investig. 2003;112:1809–1820. 14638851 10.1172/JCI20039 PMC297002

[31] Iwai-Kanai E, Yuan H, Huang C, et al A method to measure cardiac autophagic flux in vivo. Autophagy. 2008;4:322–329. 18216495 10.4161/auto.5603 PMC3709927

[32] Perry CN, Kyoi S, Hariharan N, et al Chapter 16 novel methods for measuring cardiac autophagy in vivo In: Methods in enzymology. [Internet]. 2009 [cited 2018 829]. p. 325–342. Available from: http://www.ncbi.nlm.nih.gov/pubmed/19216914 10.1016/S0076-6879(08)04016-0 PMC3658837 19216914

[33] Vanrell MC, Cueto JA, Barclay JJ, et al Polyamine depletion inhibits the autophagic response modulating Trypanosoma cruzi infectivity. Autophagy. [Internet]. 2013 [cited 2018 830];9:1080–1093. Available from: http://www.ncbi.nlm.nih.gov/pubmed/23697944 23697944 10.4161/auto.24709 PMC3722317

[34] Sbaraglini ML, Vanrell MC, Bellera CL, et al Neglected tropical protozoan diseases: drug repositioning as a rational option. Curr Top Med Chem. 2016;16:2201–2222. 26881713 10.2174/1568026616666160216154309

[35] Alba Soto CD, Mirkin GA, Solana ME, et al Trypanosoma cruzi infection modulates in vivo expression of major histocompatibility complex class II molecules on antigen-presenting cells and T-cell stimulatory activity of dendritic cells in a strain-dependent manner. Infect Immun. 2003;71:1194–1199. 12595432 10.1128/IAI.71.3.1194-1199.2003 PMC148822

[36] Melo RC, Brener Z Tissue tropism of different Trypanosoma cruzi strains. J Parasitol. [Internet]. 1978 [cited 2018 830];64:475–482. Available from: http://www.ncbi.nlm.nih.gov/pubmed/96243 96243

[37] de Diego JA, Penin P, Del Rey J, et al A comparative pathological study of three strains of Trypanosoma cruzi in an experimental model. Histol Histopathol. 1991;6:199–206. 1802119

[38] Haspel J, Shaik RS, Ifedigbo E, et al Characterization of macroautophagic flux in vivo using a leupeptin-based assay. Autophagy. 2011;7:629–642. 21460622 10.4161/auto.7.6.15100 PMC3127049

[39] Ichimura Y, Komatsu M Pathophysiological role of autophagy: lesson from autophagy-deficient mouse models. Exp Anim. 2011;60:329–345. 21791873 10.1538/expanim.60.329

[40] Eisenberg T, Knauer H, Schauer A, et al Induction of autophagy by spermidine promotes longevity. Nat Cell Biol. 2009;11:1305–1314. 19801973 10.1038/ncb1975

[41] Ramirez MI, Yamauchi LM, de Freitas LH, et al The use of the green fluorescent protein to monitor and improve transfection in Trypanosoma cruzi. Mol Biochem Parasitol. 2000;111:235–240. 11087935 10.1016/s0166-6851(00)00309-1

[42] Kayama H, Takeda K The innate immune response to Trypanosoma cruzi infection. Microbes Infect. 2010;12:511–517. 20348008 10.1016/j.micinf.2010.03.005

[43] Ray A, Dittel BN Isolation of mouse peritoneal cavity cells. J Visualized Exp. 2010;35:pii: 1488. 10.3791/1488 PMC3152216 20110936

[44] Levine B, Deretic V Unveiling the roles of autophagy in innate and adaptive immunity. Nat Rev Immunol. 2007;7:767–777. 17767194 10.1038/nri2161 PMC7097190

[45] Castillo EF, Dekonenko A, Arko-Mensah J, et al Autophagy protects against active tuberculosis by suppressing bacterial burden and inflammation. Proc Nat Acad Sci. 2012;109:E3168–E3176. 23093667 10.1073/pnas.1210500109 PMC3503152

[46] Rosado J de D, Rodriguez-Sosa M Macrophage migration inhibitory factor (MIF): a key player in protozoan infections. Int J Biol Sci. 2011;7:1239–1256. 22110378 10.7150/ijbs.7.1239 PMC3221362

[47] Chen H-R, Chuang Y-C, Chao C-H, et al Macrophage migration inhibitory factor induces vascular leakage via autophagy. Biol Open. 2015;4:244–252. 25617421 10.1242/bio.201410322 PMC4365493

[48] Vergne I, Chua J, Lee-H-H, et al Mechanism of phagolysosome biogenesis block by viable Mycobacterium tuberculosis. Proc Natl Acad Sci U S A. 2005;102:4033–4038. 15753315 10.1073/pnas.0409716102 PMC554822

[49] Gutierrez MG, Master SS, Singh SB, et al Autophagy is a defense mechanism inhibiting BCG and Mycobacterium tuberculosis survival in infected macrophages. Cell. 2004;119:753–766. 15607973 10.1016/j.cell.2004.11.038

[50] Nakagawa I, Amano A, Mizushima N, et al Autophagy defends cells against invading group A Streptococcus. Science (New York, N.Y.). 2004;306:1037–1040. 10.1126/science.1103966 15528445

[51] Sumpter R, Levine B Autophagy and innate immunity: triggering, targeting and tuning. Semin Cell Dev Biol. 2010;21:699–711. 20403453 10.1016/j.semcdb.2010.04.003 PMC2930105

[52] Lamark T, Svenning S, Johansen T Regulation of selective autophagy: the p62/SQSTM1 paradigm. Essays Biochem. 2017;61:609–624. 29233872 10.1042/EBC20170035

[53] Van Grol J, Muniz-Feliciano L, Portillo J-AC, et al CD40 induces anti-toxoplasma gondii activity in nonhematopoietic cells dependent on autophagy proteins. Urban JF, editor Infect Immun. 2013;81:2002–2011. 23509150 10.1128/IAI.01145-12 PMC3676008

[54] Koo S, Chowdhury IH, Szczesny B, et al Macrophages promote oxidative metabolism to drive nitric oxide generation in response to Trypanosoma cruzi. Appleton JA, editor Infect Immun. 2016;84:3527–3541. 27698021 10.1128/IAI.00809-16 PMC5116729

[55] Salassa BN, Romano PS Autophagy: a necessary process during the Trypanosoma cruzi life-cycle. Virulence. 2018;1–10. 10.1080/21505594.2018.1543517 PMC6550534 30489206

[56] Contreras VT, Araujo-Jorge TC, Bonaldo MC, et al Biological aspects of the Dm 28c clone of Trypanosoma cruzi after metacyclogenesis in chemically defined media. Memorias Do Instituto Oswaldo Cruz. 1988;83:123–133. 3074237 10.1590/s0074-02761988000100016

[57] Figueiredo RC, Rosa DS, Soares MJ Differentiation of Trypanosoma cruzi epimastigotes: metacyclogenesis and adhesion to substrate are triggered by nutritional stress. J Parasitol. 2000;86:1213–1218. 11191893 10.1645/0022-3395(2000)086[1213:DOTCEM]2.0.CO;2

[58] Andrade SG, Magalhães JB Biodemes and zymodemes of Trypanosoma cruzi strains: correlations with clinical data and experimental pathology. Rev Soc Bras Med Trop. 1997;30:27–35. 8993106 10.1590/s0037-86821997000100006

[59] Brener Z Therapeutic activity and criterion of cure on mice experimentally infected with Trypanosoma cruzi. Rev Inst Med Trop Sao Paulo. 1962;4:389–396. 14015230

[60] Teixeira DE, Benchimol M, Crepaldi PH, et al Interactive multimedia to teach the life cycle of Trypanosoma cruzi, the causative agent of chagas disease. Traub-Csekö YM, editor PLoS Negl Trop Dis. 2012;6:e1749. 22970330 10.1371/journal.pntd.0001749 PMC3429381

[61] Fabrino DL, Ribeiro GA, Teixeira L, et al Histological approaches to study tissue parasitism during the experimental Trypanosoma cruzi infection. Methods Mol Biol. 2011;689:69–80. 10.1007/978-1-60761-950-5_5 21153787

[62] Ni H-M, Bockus A, Wozniak AL, et al Dissecting the dynamic turnover of GFP-LC3 in the autolysosome. Autophagy. 2011;7:188–204. 21107021 10.4161/auto.7.2.14181 PMC3039769

