# Supplementary figures and images for: Autophagy plays a protective role against Trypanosoma cruzi infection in mice
Source: Virulence. 2019 Mar 4;10(1):151–65. doi: 10.1080/21505594.2019.1584027 (PMC6550547; doi:10.1080/21505594.2019.1584027)

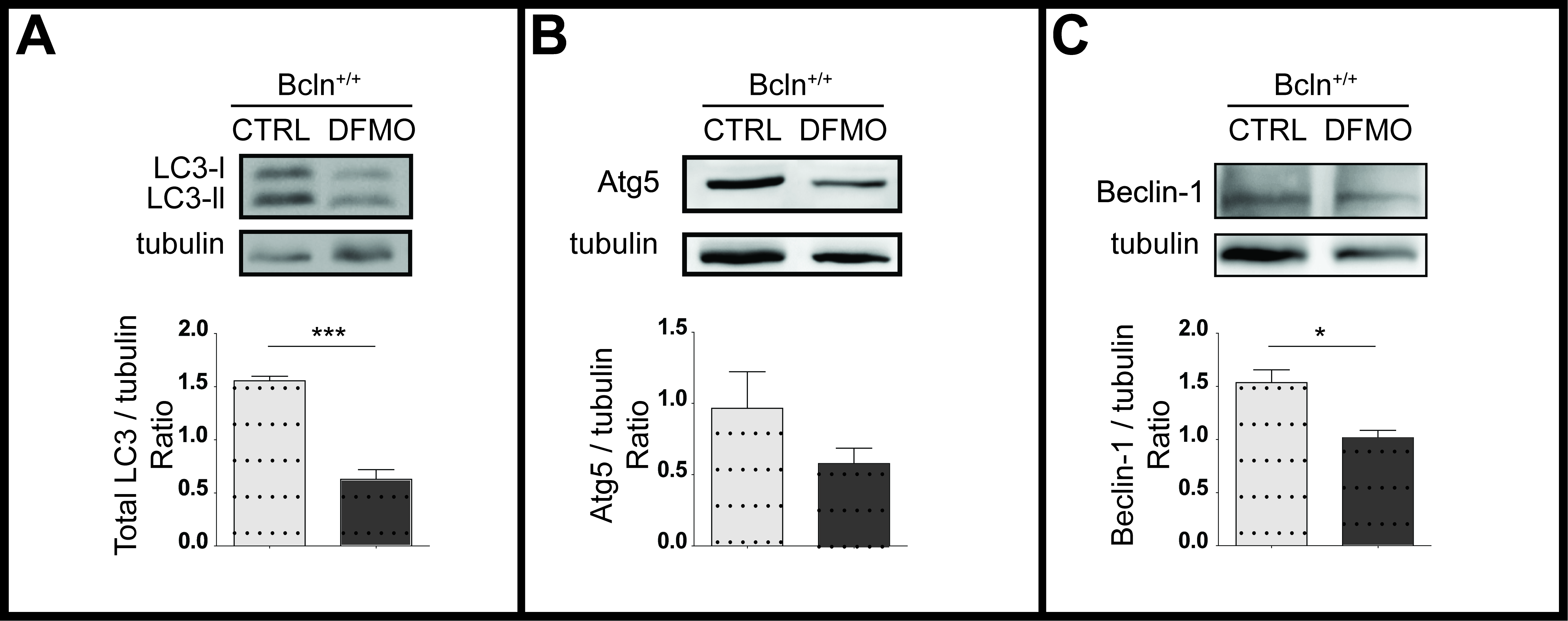

Supplement: Supplemental Material [file kvir-10-01-1584027-s001.tif]

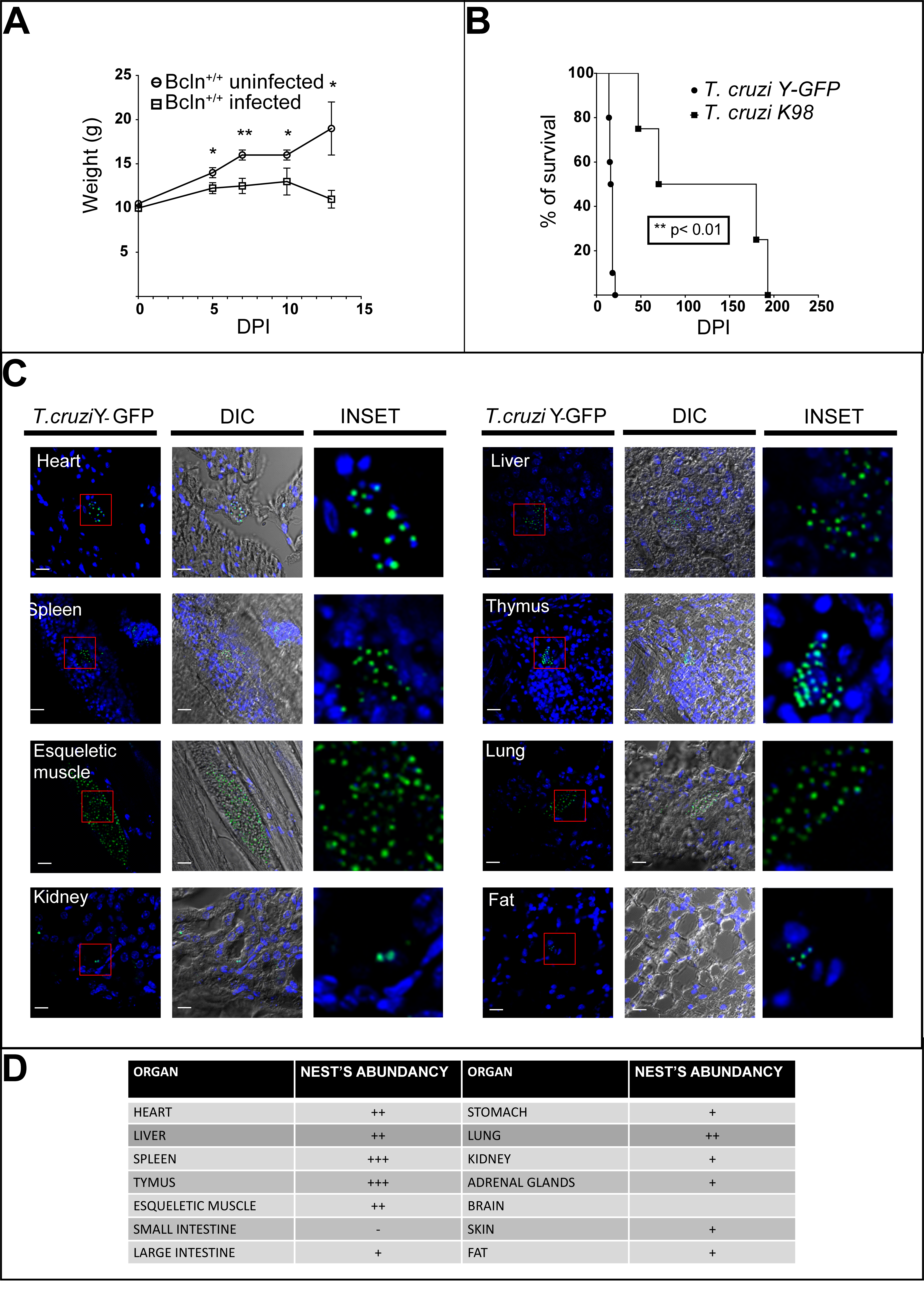

Supplement: Supplemental Material [file kvir-10-01-1584027-s002.tif]
